# Numb regulates cell tension required for mammary duct elongation

**DOI:** 10.1242/bio.042341

**Published:** 2019-04-29

**Authors:** Sudipa June Chatterjee, Ruba Halaoui, Rebecca Catherine Deagle, Carlis Rejon, Luke McCaffrey

**Affiliations:** 1 Rosalind and Morris Goodman Cancer Research Centre, McGill University, Montreal, QC H3A 1A3, Canada; 2 Division of Experimental Medicine, McGill University, Montreal, QC H4A 3J1, Canada; 3 Department of Physiology, McGill University, Montreal, QC H3G 1Y6, Canada; 4 Gerald Bronfman Department of Oncology, McGill University, Montreal, QC H4A 3T2, Canada

**Keywords:** Mammary gland, Epithelial, Morphogenesis, Tension, E-cadherin

## Abstract

The mammary gland undergoes extensive expansion of a ductal network through the stroma during puberty and is an excellent model for understanding epithelial tube morphogenesis. To investigate a role for Numb, a multifaceted adapter protein, in epithelial tube morphogenesis, we conditionally deleted it from the mammary epithelium. We report that Numb-depletion results in altered extracellular-matrix organization, reduced cell tension, altered cell shape, and increased cell packing density, which results in a 50% reduction in mammary duct elongation. Using laser ablation *in vitro* and geometric-based cell force inference *in vivo*, we determined that Numb-deficient cells have altered cortical tension. Duct elongation defects were associated with altered E-cadherin distribution, but were independent of proliferation, apoptosis in ducts or end buds. This highlights a critical role for Numb in a mechanical mechanism that is required to maintain cell packing density during epithelial tube elongation.

## Abstract (precis)

**Summary:** Numb regulates cortical cell tension, which is necessary to maintain proper cell shape and packing density during mammary duct elongation.

## INTRODUCTION

Epithelial cells can self-organize into complex structures including stratified sheets, alveolar buds and ducts, through processes of folding, budding and cavitation (Bernascone et al., 2017). The development and maintenance of epithelial tissues requires coordination of multiple processes, including apoptosis, proliferation, cell adhesion, migration and oriented cell division. Moreover, >80% of human cancers arise from epithelial cells and are characterized by disrupted epithelial architecture. Therefore, understanding the mechanisms underpinning epithelial morphogenesis may increase our understanding of carcinoma and epithelial defense against cancer.

The mammary gland is a tree-like network of epithelial ducts that produces and transports milk to feed offspring. It is composed of a polarized luminal layer with a central lumen and resides inside a myoepithelial cell layer, which is surrounded by a basement membrane (McBryan and Howlin, 2017). The mammary gland is an established model to understand the underlying mechanisms of epithelial morphogenesis. A unique advantage of the mammary gland is that the majority of its development occurs postnatally during puberty, and again during and after pregnancy (Hynes and Watson, 2010; Macias and Hinck, 2012; McBryan and Howlin, 2017).

During pubertal development ductal elongation and branching morphogenesis occurs in response to hormonal cues: estrogen, produced from the ovary, is important for the initiation and elongation of the ductal tree while progesterone promotes side branching to fill out the fat pad (Arendt and Kuperwasser, 2015; McBryan and Howlin, 2017). At the onset of puberty, specialized terminal end buds form at the ends of individual ducts in response to growth hormone, estrogen and insulin-like growth factor-1 and bifurcate to produce branches (Hynes and Watson, 2010; Macias and Hinck, 2012). End buds are a highly dynamic structure, with high proliferation and apoptosis rates, stem/progenitor activity in the cap cell layer, as well as matrix remodeling capacity as the end bud invades through the stroma (Bai and Rohrschneider, 2010; Lloyd-Lewis et al., 2017; Macias and Hinck, 2012; Paine and Lewis, 2017). In addition to growth hormone, estrogen and progesterone, various growth factors, receptors, ECM-interacting proteins, adhesion proteins, cell signaling proteins and transcription factors have been shown to regulate ductal growth and branching (McBryan and Howlin, 2017; Paine and Lewis, 2017). Indeed, mouse models reveal that that in many cases – though not all – mammary glands displayed an impaired ductal elongation phenotype that is associated with altered end bud number, morphology, or function indicating that end buds are key drivers of ductal morphogenesis and elongation during pubertal development (McBryan and Howlin, 2017). However, whereas signaling and cell dynamics during mammary gland development are becoming better appreciated (Ewald et al., 2008; Neumann et al., 2018; Paine et al., 2016; Scheele et al., 2017), we do not fully understand the biophysical, cell–cell dynamics involved in developing a mammary duct.

Numb was initially discovered as a cell fate determinant in the peripheral nervous system in *Drosophila* (Uemura et al., 1989). Subsequently, it has been implicated in stem and progenitor fate determination in various mouse tissues including cortical neurogenesis, hematopoietic stem cells, muscle progenitor cells and the mammary gland (Conboy and Rando, 2002; Rhyu et al., 1994; Uemura et al., 1989; Wakamatsu et al., 2000; Wu et al., 2007; Zhong et al., 2000; Tosoni et al., 2015; Zhang et al., 2016). Numb functions as a regulator of endocytosis of adhesion molecules like E-cadherin, and β1- and β3-integrins, which are important for epithelial cell–cell and cell–matrix interactions, respectively (Bogdanović et al., 2012; Nishimura and Kaibuchi, 2007; Sato et al., 2011; Wang et al., 2009; Zhou et al., 2011).

Targeted deletion of Numb in CK5-postive mammary basal/myoepithelial cells increases stemness of the mammary epithelial population by switching the mode of cell division from asymmetric to symmetric, which increases the stem/progenitor number (Tosoni et al., 2015). In addition, when Numb and the homolog NumbL were depleted from CK14-positive basal/myoepithelial cells, mammary glands showed a minor reduction in ductal elongation in eight-week-old mice, reduced end bud number and decreased side branching (Zhang et al., 2016). While Numb has been implicated in bi-potent progenitors, there is controversy regarding the status of multipotent mammary stem cells in the adult mammary gland, with some reports proposing that unipotent progenitors maintain distinct basal and luminal populations *in vivo* (Van Keymeulen et al., 2011; Visvader and Stingl, 2014).

The role for Numb in luminal mammary epithelial cells is unknown. To further understand how Numb regulates epithelial morphogenesis, we used MMTV-Cre to delete Numb from both luminal and myoepithelial compartments of the mammary gland. We report that deletion of Numb reduced mammary ductal length by 50% during pubertal development with associated changes in collagen organization, cell shape and cell packing density, which reveals unique functions for Numb during epithelial tube morphogenesis.

## RESULTS

### Numb is required for mammary duct elongation during puberty

**Fig. 1.**
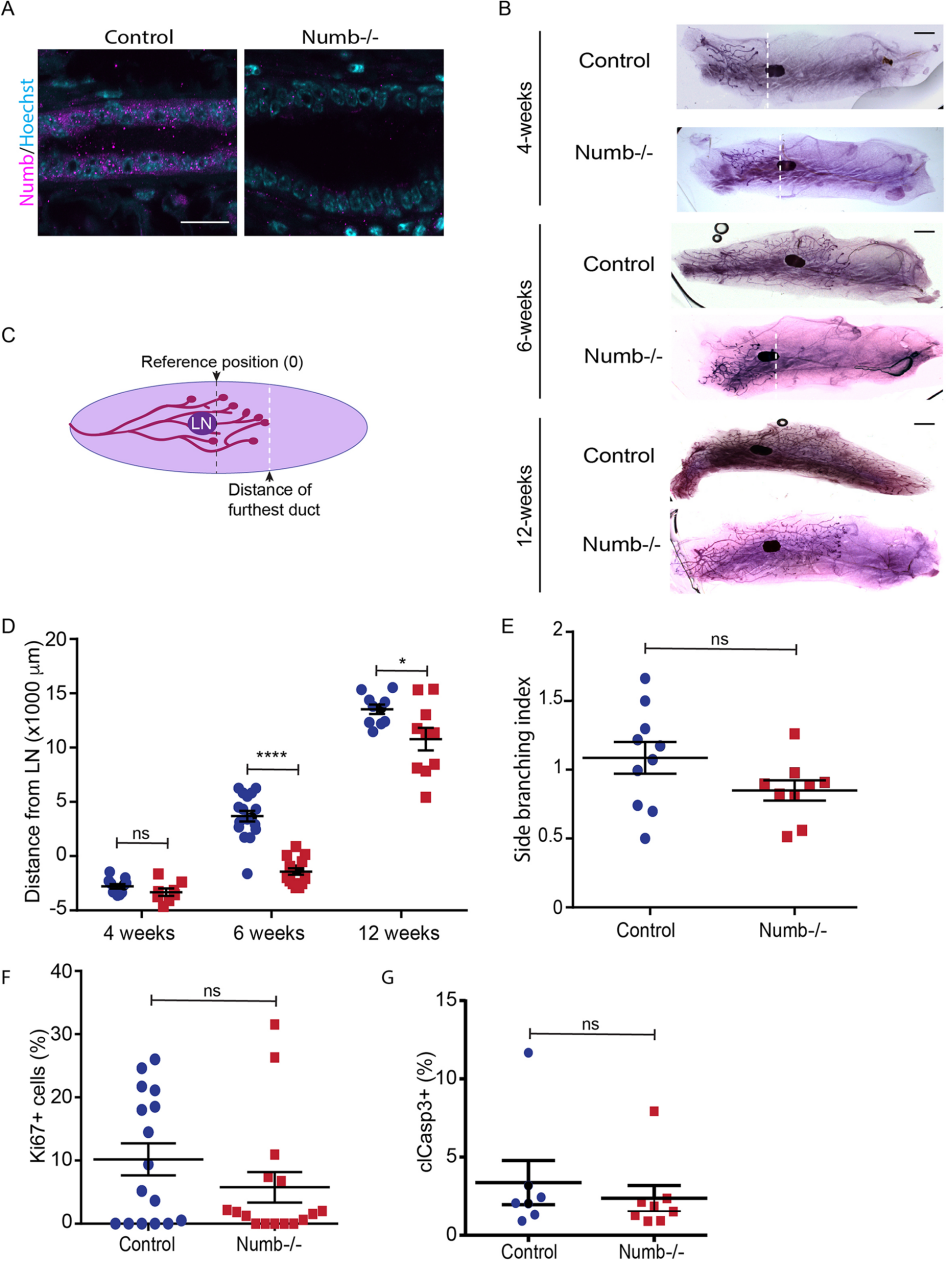
**Numb impairs ductal elongation in the pubertal mouse mammary gland.** (A) Fluorescence images of mammary ducts immunostained for Numb (magenta) in control and Numb-deficient ducts. (B) Wholemount images of control and Numb-deficient glands from four-, six- and 12-week-old mice. (C) Diagram describing growth measurements relative to the distal end of the lymph node (black dashed line, reference position). The white dashed line indicates the distal tip of the ductal tree. (D) Scatter plot of ductal outgrowth in reference to the lymph node in four-, six- and 12-week-old mice. Positive values represent growth past the distal edge of the lymph node, whereas negative values indicate ducts that have not passed the lymph node. *n*=7–15 glands for each group (one-way ANOVA). (E) Scatter plot of mammary gland branching. *n*=9–10 glands for each group. (F) Scatter plot of Ki67-positive cells in ducts. *n*=17–22 ducts from four glands for each group. (G) Scatter plot of cleaved-Caspase-3-positive cells in ducts. *n*=21 ducts from three to four glands for each group. Error bars=s.e.m. Scale bars: A, 20 μm; B, 2 mm. Unpaired *t*-test; two-tailed: ****P<0.0001; *P<0.05; ns, not significant.

To understand the function of Numb during pubertal mammary gland development we crossed mice expressing a conditional Numb allele (Numb^fl/fl^) with transgenic mice expressing Cre recombinase under the murine mammary tumor virus promoter (MMTV-Cre) (Andrechek et al., 2000), resulting in MMTV-Cre;Numb^(fl/fl)^ mice, which are Numb-deficient (Numb-/-) (Fig. 1A; Fig. S1A–C). MMTV-Cre mice were used as controls. Cre recombinase activity in both luminal and myoepithelial cells was confirmed using a tdTomato reporter strain (Tran et al., 2014), and immunostaining for CK8 and CK14 (Fig. S1D).

To evaluate a role for Numb in pubertal mammary gland morphogenesis, we examined the architecture of the wholemount inguinal gland. In six-week-old mice, control mammary ducts extend beyond the lymph node, whereas there was a 50% reduction in ductal outgrowth in Numb-deficient glands (Fig. 1B–D). By 12-weeks, all control mammary glands extended to fill the fat pad. At a similar time point, most Numb-deficient mammary glands continued to elongate and some had reached the end of fat pad (Fig. 1B,D). We conclude that Numb-deficiency induces a transient growth lag early during puberty (Fig. 1B,D). Despite reduced ductal elongation, we did not observe a statistically significant difference in the number of branches in Numb-deficient glands compared to controls (Fig. 1E).

The reduced ductal outgrowth observed in Numb-deficient mammary glands could result from impaired proliferation or survival. We investigated these possibilities by immunostaining for the proliferation marker Ki67 and the apoptosis marker cleaved-Caspase-3, but did not observe a significant difference in either proliferation or apoptosis in Numb-deficient glands compared to controls (Fig. 1F,G; Fig. S1E). Therefore, we conclude that Numb is required for ductal elongation during early pubertal development.

### Numb does not affect mammary end bud structure

**Fig. 2.**
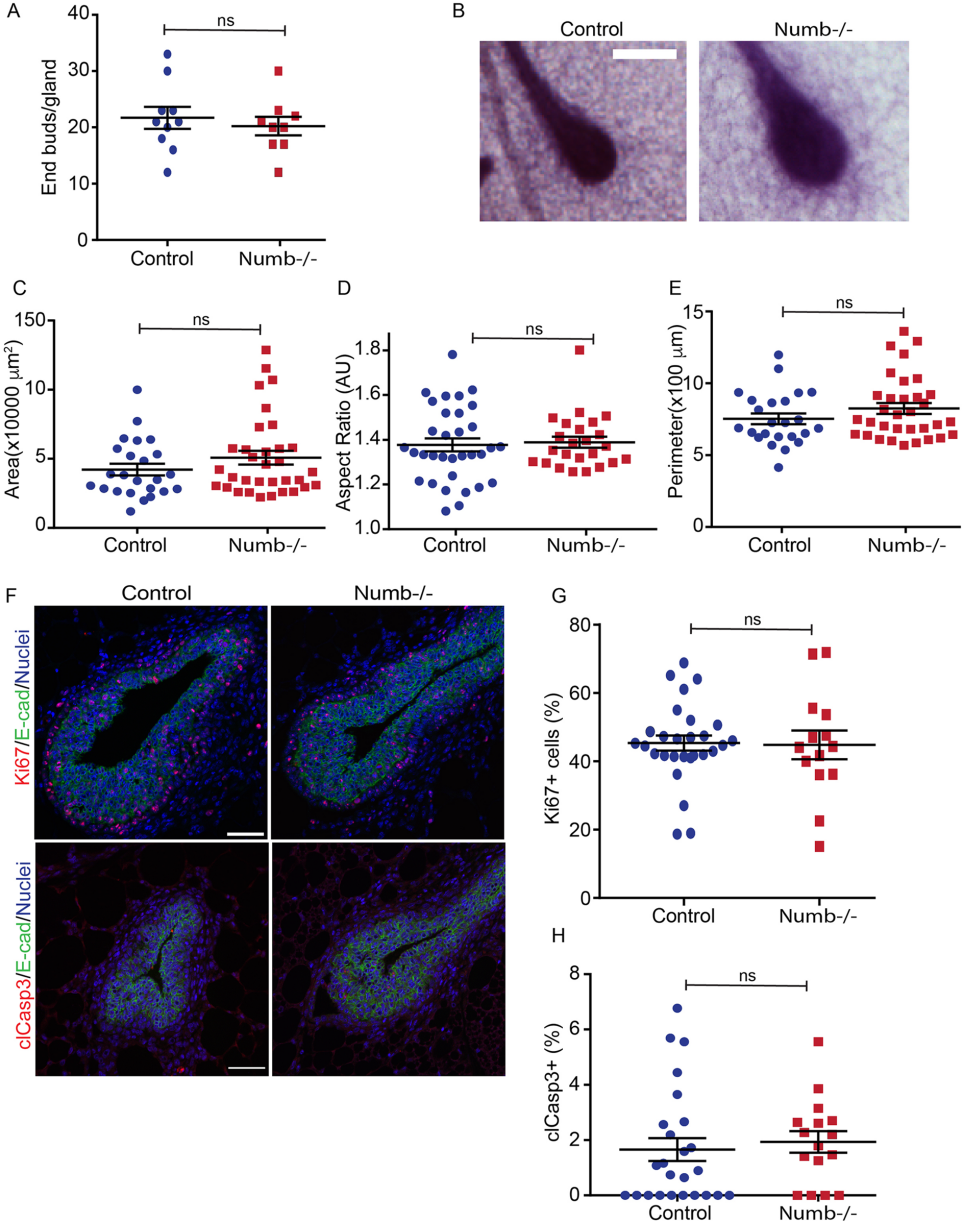
**Loss of Numb does not affect mammary end bud structure.** (A) Scatter plot of the number of end buds in control and Numb-deficient mammary glands. *n*=10 glands for each group. (B) Images of end buds from mammary gland wholemounts. (C) Scatter plot of end bud area. (D) Scatter plot of end bud aspect ratio (length/width). (E) Scatter plot of end bud perimeter. (F) Fluorescence images of end buds immunostained for Ki67 (proliferation) and cleaved-Caspase-3 (apoptosis). (G) Scatter plot of Ki67-positive cells in end buds. (H) Scatter plot of cleaved-Caspase-3-positive cells in end buds. Error bars=s.e.m. Scale bars: B, 250 μm; F, 50 μm. Unpaired *t*-test; two-tailed: ns, not significant. *n*=24–33 end buds from six to eight glands per group (C–E); *n*=16–26 ducts from five to 11 glands per group (G,H).

Terminal end buds promote mammary ductal growth during puberty, and defects in end bud function have been reported to impair ductal elongation (McBryan and Howlin, 2017; Paine and Lewis, 2017). We therefore examined if end bud number or morphology were altered in Numb-deficient glands. In mammary glands from six-week-old mice, we found that end buds from Numb-deficient mammary glands were indistinguishable from controls in terms of number, size and shape (Fig. 2A–E). End buds are highly proliferative, and provide cells to be deposited into ducts, while apoptosis contributes to lumen formation (Mailleux et al., 2008; Paine and Lewis, 2017). We did not observe a difference in either proliferation or apoptosis between Numb-deficient and control end buds, consistent with no difference observed in ducts (Fig. 2F–H). Collectively, these data indicate that the delay in ductal elongation in Numb-deficient mammary glands is not associated with changes in proliferation or survival in the ducts or end buds.

### Peri-epithelial collagen organization is altered in Numb-deficient mammary glands

**Fig. 3.**
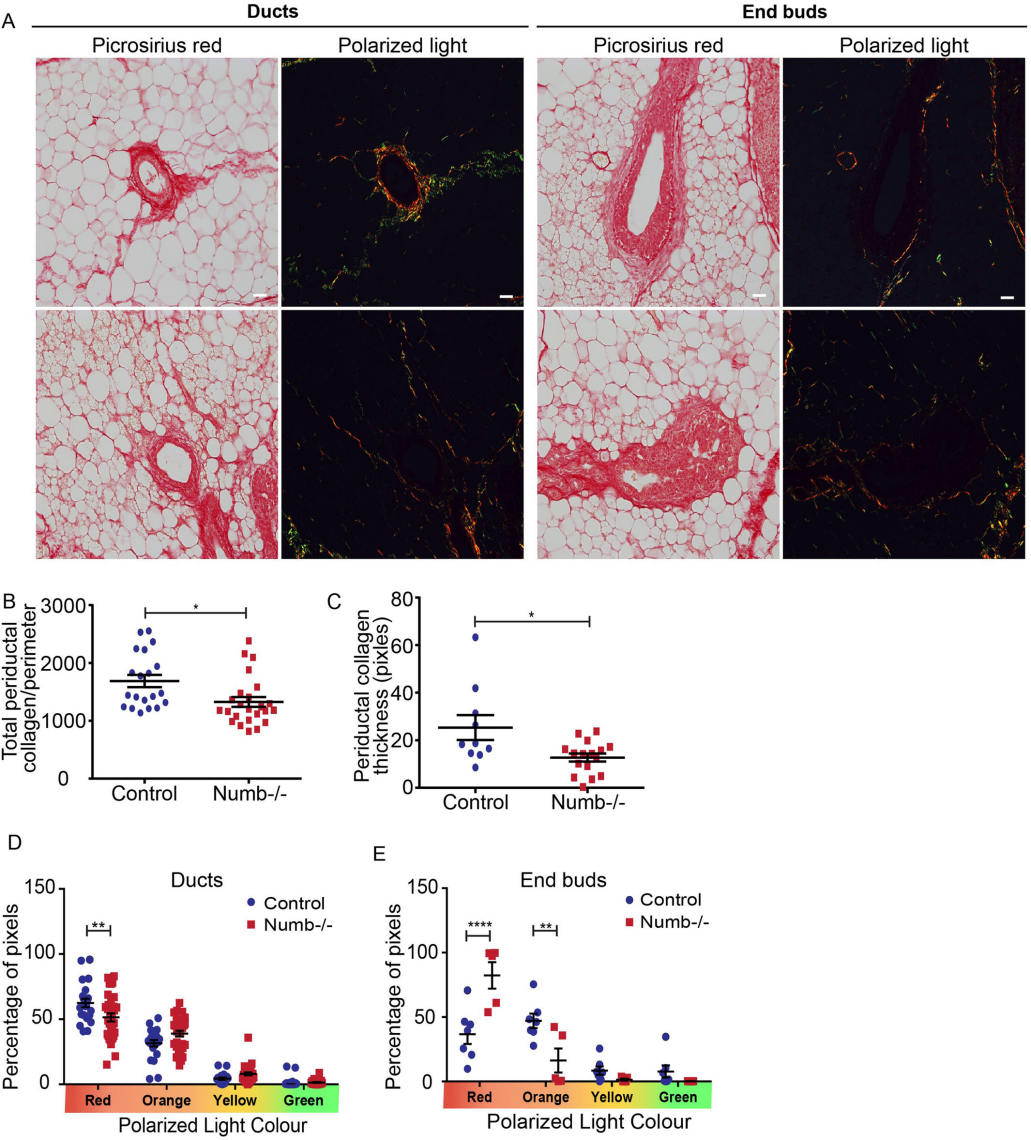
**Density and organization of collagen is affected by Numb.** (A) Brightfield and polarized light images of Picrosirius Red stained mammary tissue sections from control and Numb-deficient mice. (B) Scatter plot of total periductal collagen normalized to the perimeter of the duct from polarized light images. *n*=20–24 ducts from five to six glands for each group. (C) Scatter plot of the mean thickness (width) of collagen around ducts from polarized light images. *n*=13–16 duct from four glands for each group. (D) Quantification of the proportion of pixels binned into red, orange, yellow and green categories from polarized images of periductal collagen. *n*=22–33 ducts from six to seven glands for each group (multiple *t*-test). (E) Quantification of the proportion of pixels binned into red, orange, yellow and green from polarized light images of collagen surrounding control and Numb-deficient end buds. *n*=5–7 end buds from three to five glands (multiple *t*-test). Error bars=s.e.m. Unpaired *t*-test; two-tailed: ****P<0.0001, **P<0.01, *P<0.05. Scale bar: 50 μm.

Recent studies indicate that epithelial-stromal interactions involving collagen I are important regulators of pubertal mammary morphogenesis (McBryan and Howlin, 2017; Paine and Lewis, 2017; Hammer et al., 2017; Nguyen-Ngoc and Ewald, 2013; Peuhu et al., 2017). The anisotropy of collagen structure reflects its organization (Rittie, 2017). Birefringence measurements using polarized light microscopy can distinguish regions of thin, loose collagen bundles from denser bundles, based on color in the green-red spectrum (Arun Gopinathan et al., 2015; Devendra et al., 2018; Drifka et al., 2016). Numb-deficient glands had overall less collagen around the epithelium (Fig. 3A–C) and a relatively modest, but statistically significant, reduction in collagen dense bundles around duct regions (Fig. 3D, reduced red signal of Numb-/-). However, there was a dramatic increase in collagen density surrounding end bud from Numb-deficient mammary glands (Fig. 3E, increased red signal of Numb-/-).

### Numb-deficiency disrupts epithelial cell morphology and packing density

**Fig. 4.**
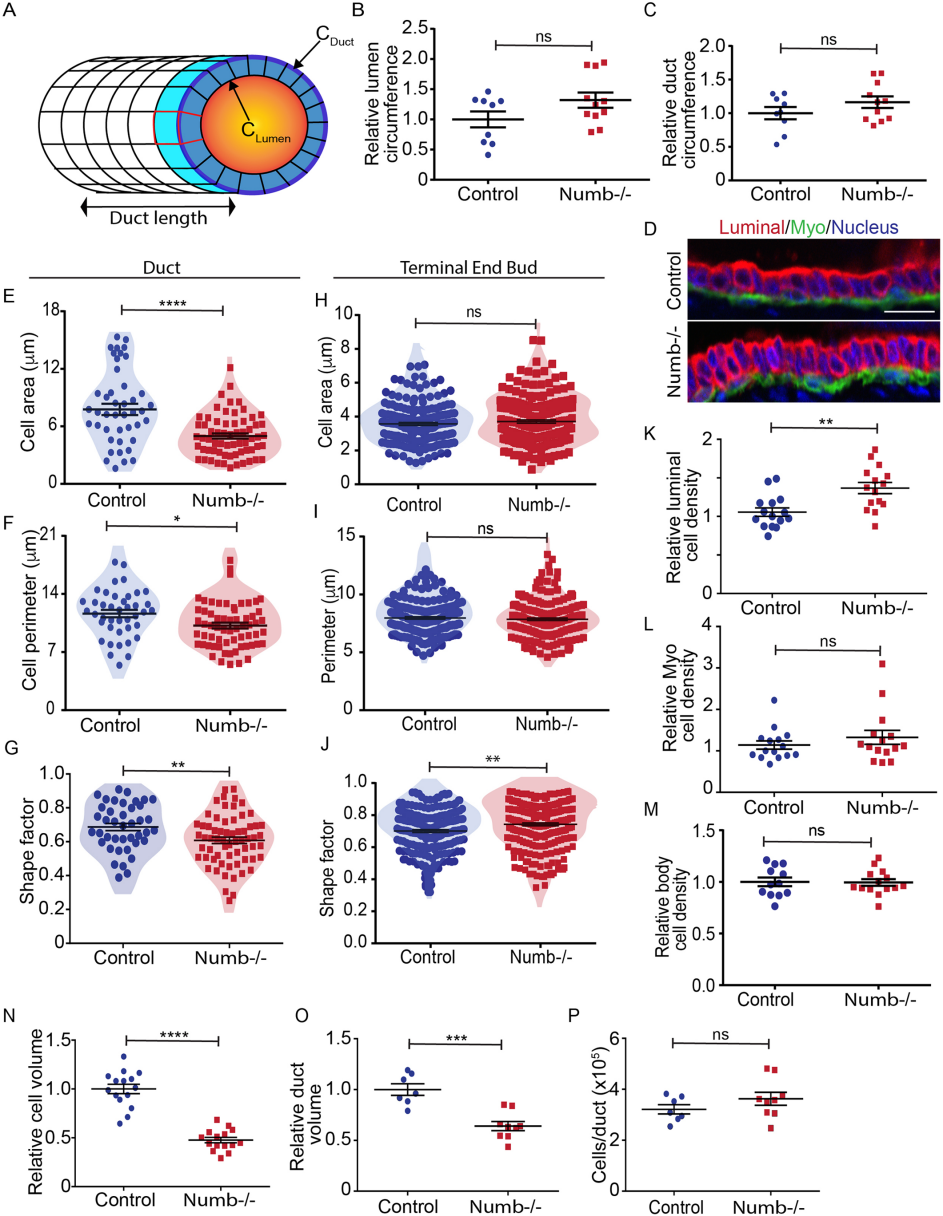
**Numb affects luminal cell shape in pubertal mammary glands.** (A) Diagram of a duct showing measurement parameters: C_lumen_ represents the inner circumference of the lumen; C_duct_ represents the outer circumference of the duct; dark blue represents the cross-sectional face of the duct; light blue represents the cell length along the duct. Red represents the outline of a luminal cell. (B) Scatter plot of lumen size (C_lumen_) for control and Numb-depleted mammary ducts_._ (C) Scatter plot of duct size (C_duct_) for control and Numb-depleted mammary ducts. (D) Fluorescence images of luminal (red) and myoepithelial (Myo; green) cell markers in control and Numb-deficient ducts. Scale bar: 20 μm. (E) Scatter plot of luminal duct cell areas. Color shading on the scatter plots shows the density distribution of data. (F) Scatter plot of luminal cell perimeters. (G) Scatter plot of luminal cell shapes. Shape factor varies from circular (0) to elongated polygon (1.0). (H) Scatter plot of end bud body cell areas. (I) Scatter plot of end bud body cell perimeters. (J) Scatter plot of end bud body cell shapes. (K) Scatter plot of cell packing density (number of cells/unit length) for luminal duct cells. (L) Scatter plot of cell packing density for myoepithelial (Myo) duct cells. (M) Scatter plot of cell packing density for end bud body cells. *n*=12–14 ducts over four glands for each group. (N) Scatter plot showing calculated average ductal cell volumes. *n*=14 cells over four to five glands for each group. (O) Scatter plot showing calculated relative volume of mammary ducts. (P) Scatter plot showing calculated total number of cells per duct. Error bars=s.e.m. Unpaired *t*-test; two-tailed: ****P<0.0001, ***P<0.0005, **P<0.01, *P<0.05, ns, not significant. *n*=9–11 ducts from five to six glands for each group (B,C); *n*=43–66 cells from three glands for each group (E–G); *n*=187–216 cells from four glands for each group (H–J); *n*=15 ducts over five glands for each group (K,L); *n*=7–9 ducts over seven to nine glands for each group (O,P).

Since we did not observe major discrepancies in ends buds between Numb-deficient and control glands, we examined the ducts more closely to determine if there were features that could contribute to reduced elongation. One possibility was that Numb-deficient duct geometry could be altered, such that growth occurs to increase duct diameter at the expense of elongation. From confocal images of tissue cross-sections, we measured the duct circumference (C_duct_) and the width of luminal cavity (C_lumen_), but found that there was no statistically significant difference in either circumference (Fig. 4A–C). This is consistent with the wholemounts, where no obvious difference in duct width was observed (Fig. 1).

Since Numb has been reported to regulate mammary stem cell divisions (Tosoni et al., 2015), we examined luminal and myoepithelial lineages by immunostaining for CK8 and CK14, respectively. CK8/CK14-double positive cells have been suggested as a bi-potent progenitor population (Sun et al., 2010). However, we did not observe rare CK8/CK14 double positive cells in either Numb-deficient and control glands. Instead, both markers were restricted to luminal and myoepithelial cell types, which were present as an intact bilayer (Fig. 4D). However, we observed striking differences in the organization of luminal epithelial cells in ducts. Compared to cells from control ducts, Numb-deficient cells appeared narrower and more compact compared to cuboidal-shaped luminal cells found in control glands (Fig. 4D). To quantify these apparent differences, we measured multiple shape descriptors of individual cells and found that indeed Numb-deficient cells had a smaller area, perimeter, and were shifted towards an elongated polygon shape, compared to control cells (Fig. 4E–G). Furthermore, we quantified cell density along ducts, i.e. the number of luminal or myoepithelial cells per unit length of duct. Remarkably, we observed a 36% increase in packing of luminal cells in Numb-deficient ducts compared to control ducts; however, the myoepithelial cell packing was unaffected (Fig. 4K,L). We next examined whether cell geometry was altered in end bud body cells or cap cells, the precursors of luminal and myoepithelial cells in the subtending ducts. Although there was a modest, but statistically significant change in shape, the cross-sectional area of cells was not different between Numb-deficient and control end bud body cells (Fig. 4H–J). Interestingly, body cell density in the end bud was not different between Numb-deficient and control end buds (Fig. 4M). Therefore, Numb controls epithelial cell shape and affects packing density specifically in the luminal epithelial compartment of developing mammary ducts.

We next asked if the tighter cell packing density could account for the reduced mammary duct length observed in six-week-old glands. To address this, we estimated the total number of cells in ducts from Numb-deficient and control mammary glands by measuring the dimensions of cells from sagittal and cross-sections from confocal images. For both control and Numb-deficient we calculated that the average Numb-deficient cell occupies half the space as a control cell (Fig. 4N). For comparisons, we restricted our analysis to the longest duct in each gland. Using length measurements of the longest duct from wholemount mammary glands, and the ductal cross-sectional area, we estimated the cylindrical volume of each duct (Fig. 4O); dividing this by the space occupied by individual cells yielded an estimate of the total number of cells in the longest duct. Remarkably, we found that the total number of cells in the longest mammary ducts was similar between Numb-deficient and controls (Fig. 4P). Therefore, we conclude that increased cell packing density is likely a major contributor to reduced duct elongation in pubertal Numb-deficient mice.

### Effect of Numb-deficiency on duct elongation is epithelial autonomous

**Fig. 5.**
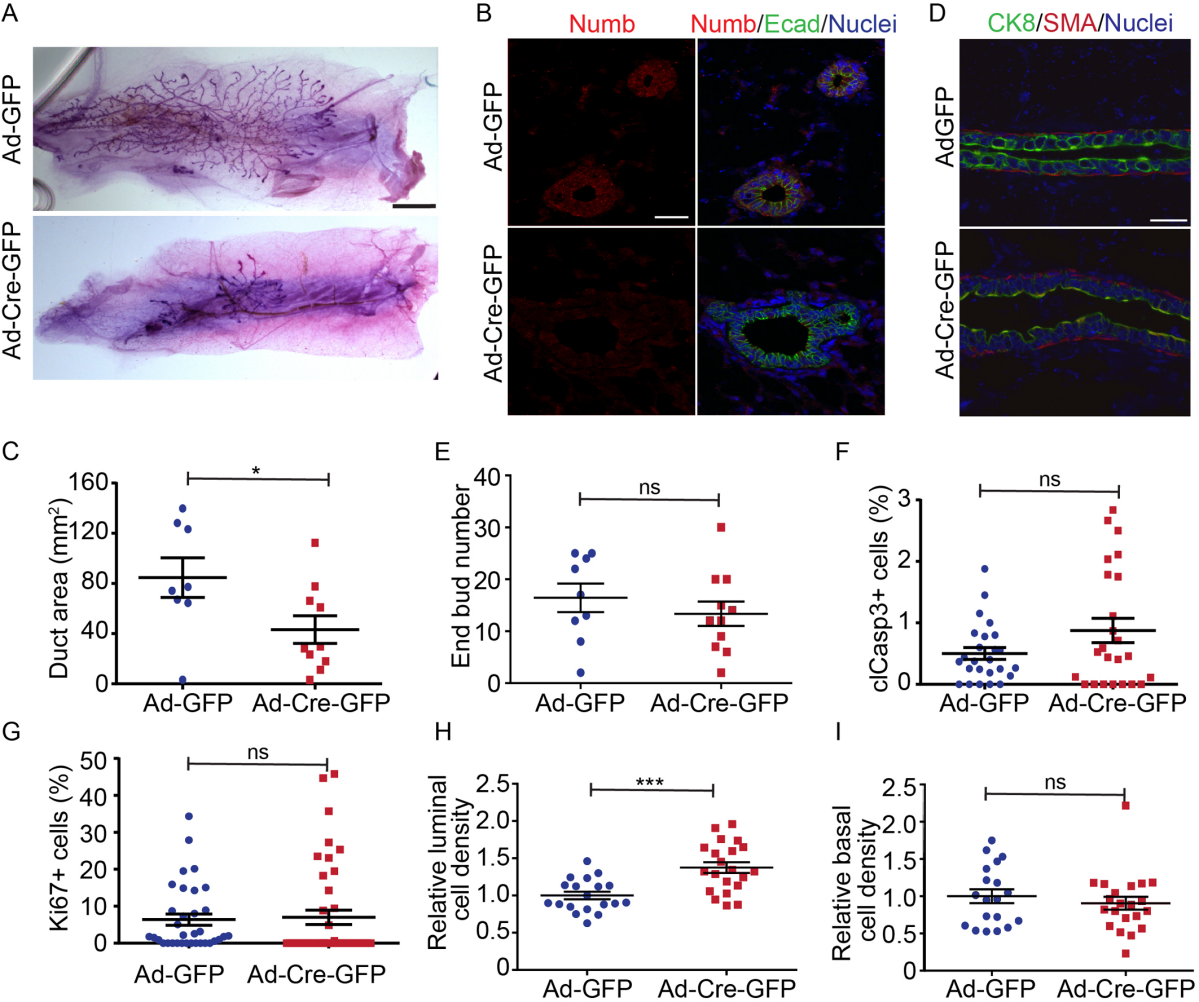
**Numb phenotype is autonomous to the epithelium.** (A) Images of mammary gland wholemounts of control (Ad-GFP) and Numb-deficient (Ad-Cre-GFP) mammary fat pad transplants. (B) Fluorescence images of transplanted mammary glands immunostained for Numb (red) and E-cadherin (green). (C) Scatter plot of the total area of ductal coverage of fat pad transplants. (D) Fluorescence images of mammary transplants immunostained for CK8 and SMA to show luminal and myoepithelial cells, respectively. (E) Scatter plot of the total number of end buds from transplant outgrowths. (F) Scatter plot of cleaved-Caspase-3-positive cells from transplant outgrowths. *n*=24–25 ducts from eight to ten glands for each group. (G) Scatter plot of Ki67-positive cells from transplant outgrowths. *n*=34–43 ducts from eight to ten glands for each group. (H) Scatter plot showing the relative luminal cell density (number of cells/unit length). (I) Scatter plot showing the relative myoepithelial cell density. *n*=8–10 glands for each group (C,E). *n*=19–21 ducts from eight to ten glands for each group (H,I). Error bars=s.e.m. Scale bars: B, 2000 μm; C, 20 μm; D, 20 μm. Unpaired *t*-test; two-tailed: ***P<0.0005, *P<0.05, ns, not significant.

Pubertal mammary gland development is initiated by systemic hormones and stromal growth factors. Moreover, MMTV has been reported to express in tissues other than the mammary gland (Andrechek et al., 2000). Therefore, to determine whether the Numb-deficient phenotype was mammary epithelial intrinsic we performed mammary fat pat transplants. For this we isolated cells from Numb^(fl/fl)^ mice and infected them *ex vivo* with adenovirus expressing Cre-recombinase fused to GFP (Ad-Cre-GFP), or GFP alone (Ad-GFP) as a control, then transplanted unsorted cells into the cleared fat pads of three-week-old recipient mice. Five weeks post-transplantation, mammary fat pads were isolated and ductal outgrowths were visualized by wholemount staining (Fig. 5A). The transplant efficiency was similar for both Ad-Cre-GFP (10/10) and Ad-GFP (8/10) transplants, and we confirmed Numb loss by immunofluorescence staining (Fig. 5B). However, the extent of the mammary gland outgrowths from Numb-deficient cells were significantly smaller than control transplants (Fig. 5A,C). Outgrowths formed end buds, displayed luminal/myoepithelial bilayers, and did not display differences in proliferation or apoptosis between Numb-deficient and control (Fig. 5D–G). Furthermore, we observed that the cell packing density of luminal, but not myoepithelial cells, was higher in Numb-deficient transplants compared to controls (Fig. 5H,I). Therefore, the cell phenotypes were similar between transplanted mammary outgrowths and our MMTV-Numb^(fl/fl)^ transgenic mouse model and indicate that these phenotypes are epithelial autonomous.

### E-cadherin distribution is altered in Numb-deficient luminal epithelial cells

**Fig. 6.**
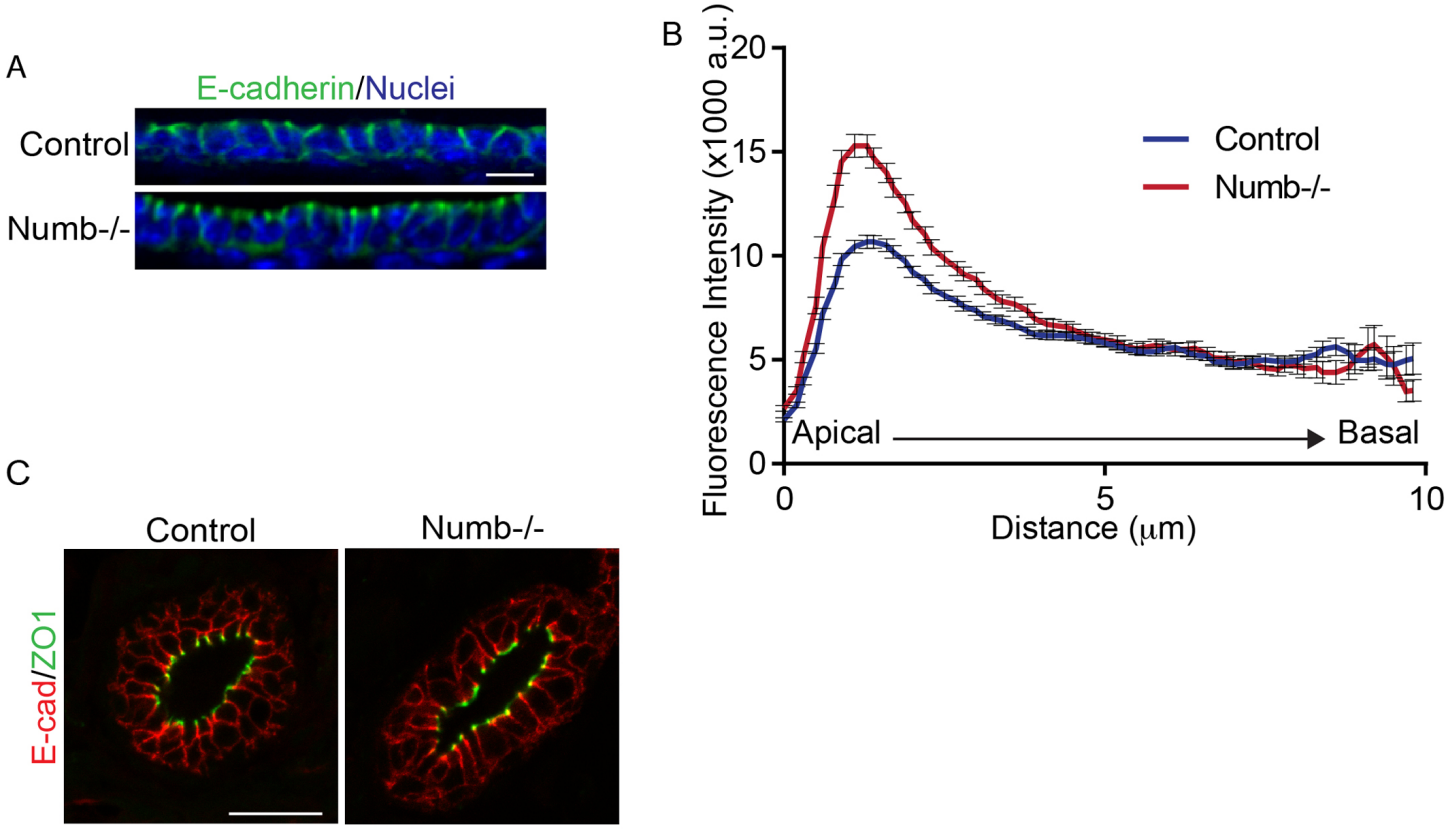
**Numb affects E-cadherin localization at adherens junctions.** (A) Fluorescence images of control and Numb-deficient ducts immunostained for E-cadherin (green). (B) Line tracing of E-cadherin fluorescence intensity along lateral membrane from apical to basal (*P*<0.0001). *n*=121–185 cells analyzed from three to four glands from both groups (paired *t*-test; two-tailed). (C) Fluorescence images of control and Numb-deficient ducts immunostained for tight junction marker ZO1 (green) and E-cadherin (red). Error bars=s.e.m. Scale bars: A, 20 μm; C, 20 μm.

Cell adhesions play essential roles in regulating epithelial cell morphology (Takeichi, 2014). Previous studies in cultured cells indicate that Numb can regulate cell adhesions by controlling endocytosis of E-cadherin and β1-integrin (Sato et al., 2011; Nishimura and Kaibuchi, 2007; Bogdanović et al., 2012). We therefore examined whether localization of these proteins was altered in Numb-deficient mammary epithelium. In both Numb-deficient and control mammary glands, E-cadherin was localized to the basolateral domains. However, we observed a 1.5-fold enrichment of E-cadherin to the apical side of the lateral membrane (Fig. 6A,B). There was no apparent effect on tight junctions (Fig. 6C). Unlike E-cadherin, no differences in β1-integrin localization were observed between control and Numb-deficient ducts (Fig. S2A,B). Our data support that E-cadherin recycling is mediated by Numb during pubertal mammary gland morphogenesis.

### Numb-deficiency causes surface disorganization but does not affect branching in organoid cultures

**Fig. 7.**
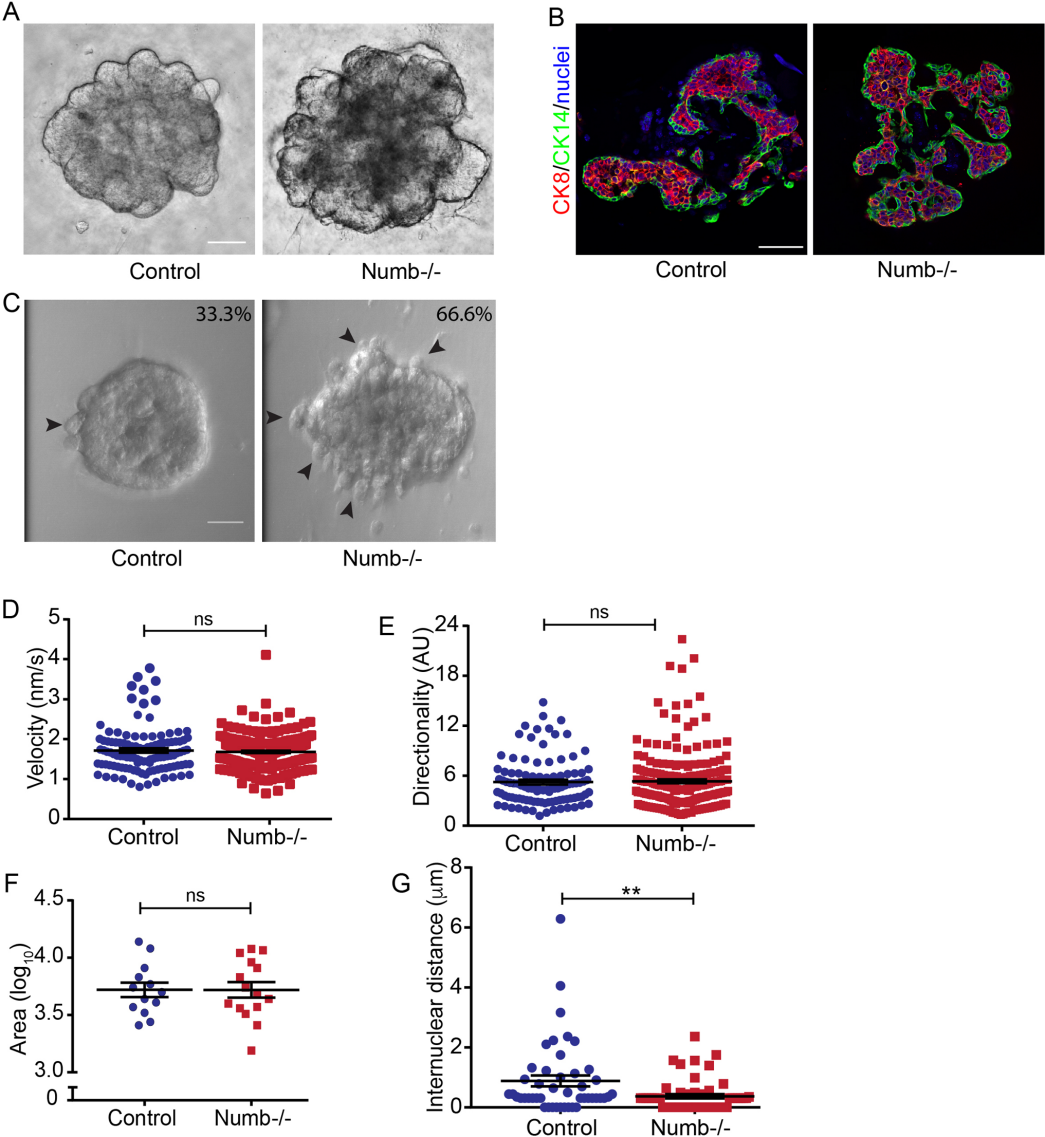
**Numb is not required for epithelial cell migration during organoid morphogenesis.** (A) DIC images of control and Numb-deficient organoids after 3 days in culture. (B) Fluorescence images of sections of control and Numb-deficient organoids immunostained for CK8 and CK14 and label luminal and myoepithelial lineages. (C) DIC images of control and Numb-deficient organoids. Numbers and the percentage of organoids showing surface disorganization (see arrowheads). (D) Scatter plot of average cell velocity in control and Numb-deficient organoids. (E) Scatter plot of cell migration directionality (ratio of total distance over displacement). (F) Scatter plot of total organoid area from control and Numb-deficient organoids. *n*=13–15 organoids from both groups. (G) Scatter plot of cell packing density measured from internuclear distances of cells in control and Numb-deficient organoids. *n*=46–54 neighboring cell pairs from five to 11 organoids from both groups. *n*=100–215 cells from nine to 13 organoids from both groups (D,E). Error bars=s.e.m. Scale bars: 50 μm; Unpaired *t*-test; two-tailed: **P<0.01; ns, not significant.

Cell migration dynamics are required for mammary duct morphogenesis and elongation (Ewald et al., 2008; Neumann et al., 2018). To investigate if Numb-depletion affected cell migration, we cultured mammary organoids from Numb-deficient and control mice. Lentivirus was used to express mCherry-histone to better visualize nuclei to track cell movements. Numb-depleted organoids were able to branch in culture, like controls (Fig. 7A,B). However, 66.6% of Numb-deficient organoids exhibited surface disorganization compared to the 33.3% of controls (66 Numb-/- and 48 control organoids imaged) (Fig. 7C). Consistent with no impairment in branching, there were no differences in average cell velocity or directionality of motion between organoids from Numb-deficient and control mice (Fig. 7D,E). Despite no changes in migration per se, we noted that like the *in vivo* results above, the cell packing density was increased in organoids from Numb-deficient mice (Fig. 7F). Therefore, we conclude that Numb does not directly regulate cell migration required for mammary morphogenesis.

### Numb-deficient cells display altered epithelial cell tension

**Fig. 8.**
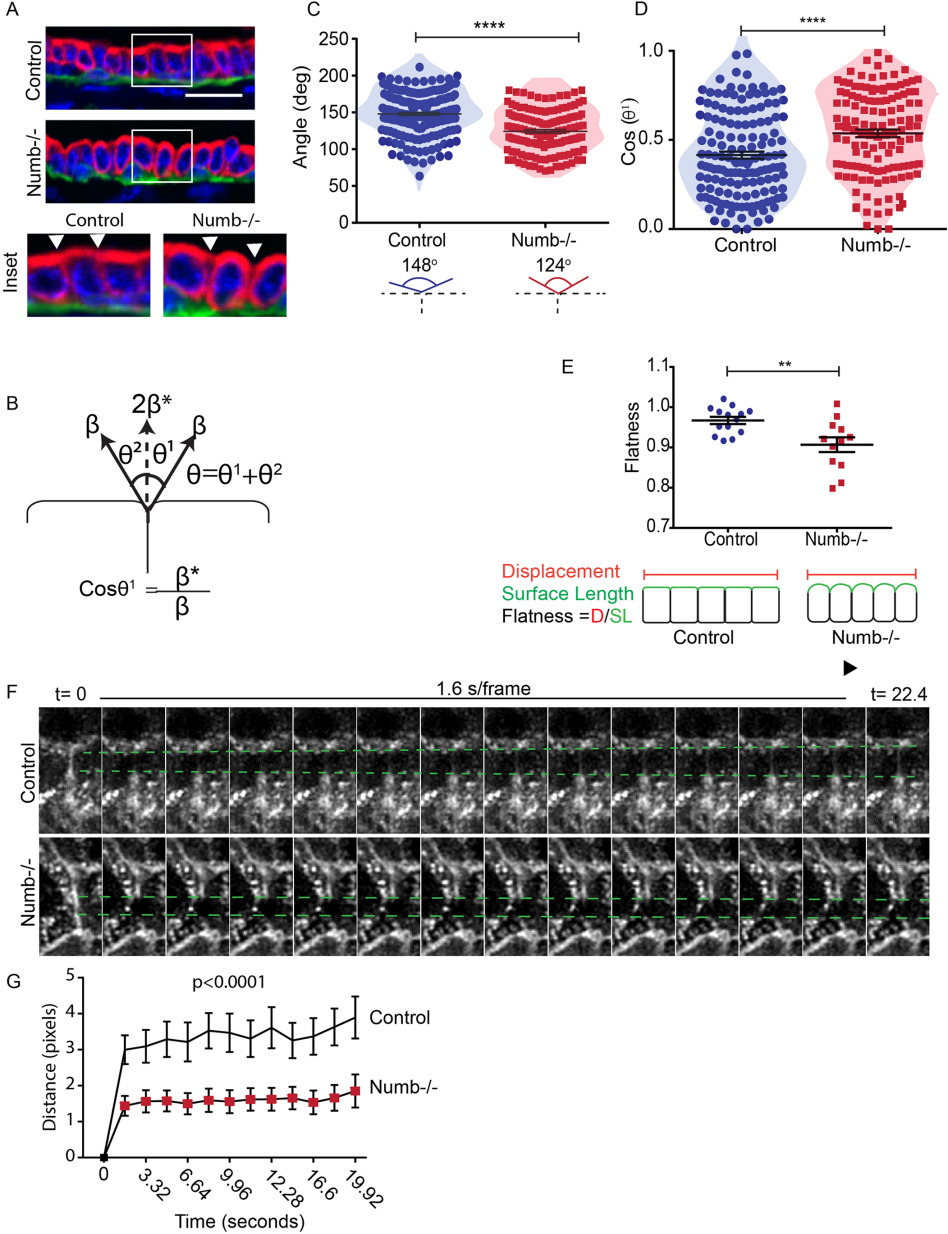
**Numb regulates cell tension in mammary epithelial cells.** (A) Fluorescence of images of control and Numb-deficient mammary glands immunostained for CK8 (red). White arrowheads indicate points of cell–cell contact. (B) Diagram showing measured angles between adjacent cells: β, cortical tension at the free surface relates to β*, the tension at cell–cell contacts, through cosine of the angle between them. (C) Scatter plot of θ between adjacent control or Numb-deficient cells. *n*=140–179 neighboring cells. (D) Scatter plot of Cos(θ^1^) for control and Numb-deficient cells. *n*=130–147 cells. (E) Scatter plot of the epithelial duct flatness along the apical surface. 12–14 ducts from both groups. (F) Time-lapse images of a control and Numb-deficient before (t=0) and after laser ablation. Green dashed lines indicated the deviation of the membranes from the t=0 position after ablation. (G) Quantification of the recoil distance following laser cutting of mammary epithelial cells (*P*<0.0001). *n*=25–27 cells from both groups (paired *t*-test, two-tailed). Error bars=s.e.m. Unpaired *t*-test; two-tailed: ****P<0.0001, **P<0.01. Scale bar: 20 μm.

The increased surface disorganization of Numb-deficient organoids suggested to us that cortical cell tension may be disrupted in these cells. Moreover, cell shape is influenced by cortical cell tension conferred intracellularly through the cytoskeleton and externally through interactions with the extracellular matrix and adhesions with neighboring cells (Diz-Muñoz et al., 2013) and tension contributes to duct elongation (Neumann et al., 2018). We observed that the apical surface of the Numb-null ducts appeared uneven compared to the much flatter control surface (Fig. 8A). This prompted us to consider whether cell tension was altered in Numb-deficient glands. The angle (θ^1^) depends on the cortical tension at the unattached apical surface (β) and to the tension at cell–cell contacts (β*) (Fig. 8B) (David et al., 2014; Winklbauer, 2015). We therefore used this relationship to assess the possibility that cell tension was altered in Numb-depleted mammary gland cells. We measured the combined angle between two adjacent cells (θ) and found that control cells had a larger angle compared Numb-deficient cells (Fig. 8C). Cos(θ^1^) represents the ratio of tension at cell contacts to tension at the free cell surface (David et al., 2014; Winklbauer, 2015) between a value between 0 and 1. Numb-deficient cells have a higher cos(θ^1^) ratio, indicating a shift in the balance of cortical tension between the free surface and the cell–cell contacts (Fig. 8D). To further examine the change in tension ratio along the entire duct in relation to cell packing density, we measured the degree of flatness as a ratio of surface length to displacement (Fig. 8E). A flatness of 1 indicates a flat planar apical membrane along a duct, whereas a shift away from 1 indicates a curved surface consistent with tension differences. We observed that Numb-deficient ducts had a significantly reduced flatness factor compared to control, which supports that the changes in apical-to-cell–cell tension manifests globally (Fig. 8E).

To provide additional evidence for a role for Numb in regulating cortical cell tension in mammary epithelial cells, we performed laser ablation at cortical sites of cell–cell contact. Following laser cutting, the recoil response is a function of the junctional tension (Liang et al., 2016). We observed that the average recoil distance was significantly higher in control cells compared to Numb-deficient cells (Fig. 8F,G). We therefore conclude that Numb alters cortical tension in mammary epithelial cells, indicating that Numb-deficient cells have less tension across their membranes.

## DISCUSSION

We report that Numb is required for pubertal mammary gland development. In MMTV-Cre/Numb^fl/fl^ mice, the major phenotype we observed is a transient reduction in the extension of ducts. The apparent shortening of mammary ducts could result from either reduced ductal elongation or increased tortuosity, caused by more frequent branching (Paine et al., 2016). For example, loss of Plk2 results in a transient delay in the time for mammary ducts to reach the end of the fat pad during puberty, but with increased proliferation and branching, suggesting that duct elongation per se was not be affected, but the pattern of ductal growth is altered between the balance of branching and lengthening (Villegas et al., 2014). Despite a transient reduction in the displacement length of Numb-deficient mammary glands, we did not observe an increase in branching or duct diameter, supporting that the defects are directly due to duct elongation. Moreover, our measurements of individual ductal cells show that they are narrower than control cells, resulting in an increased cell packing density. Importantly, we did not observe significant changes in proliferation or apoptosis in Numb-deficient mammary glands. To further support that cell packing density is altered independently of cell number, we made detailed measurements to estimate the number of cells in a single mammary duct. Remarkably, Numb-deficient mammary ducts had the same number of cells but were ∼50% shorter than control ducts at 6 weeks of age.

Pubertal mammary gland development initiates with hormonal regulation of proliferation and epithelial remodeling. At the onset, terminal end buds develop which undergo extensive proliferation to provide sufficient cells to elaborate an epithelial ductal tree that extends from a rudimentary structure to fill the fat pad (Huebner and Ewald, 2014; McBryan and Howlin, 2017; Paine et al., 2016; Paine and Lewis, 2017; Scheele et al., 2017). Several studies have correlated end bud size and structure with mammary ductal outgrowth during pubertal development. For example, studies on mosaic FGFR2 in the mammary gland revealed a proliferation disadvantage of FGFR2-null in end buds, with concomitant reduced ductal length transiently during pubertal development (Lu et al., 2008). Defects in end bud morphology in ErbB2- or ErbB3-null mammary glands were also associated with reduced ductal growth (Jackson-Fisher et al., 2008; Jackson-Fisher et al., 2004). In contrast, we did not observe differences in end bud morphology, proliferation, or apoptosis, indicating that Numb unlikely impairs end bud directly during pubertal mammary gland development.

Recent elegant studies using computer modeling, organoid cultures, and *in vivo* lineage tracing have described that end bud body cells rearrange and migrate to generate the subtending duct, whereas epithelial cells in mature ducts do not migrate (Neumann et al., 2018; Paine et al., 2016; Scheele et al., 2017). Neumann et al. found that migratory cells form protrusions which show increased Ras and PI3K activity and the presence of polymerized F-actin, allowing luminal cells to intercalate at the basal surface and expand the surface area causing ductal elongation, and effectively linking cell migration to ductal elongation (Neumann et al., 2018; Scheele et al., 2017). Considering that our analysis showed no differences in end bud morphology, Numb could instead be involved in regulating cell dynamics during coalescing end buds into ducts. However, we found that Numb-deficient mammary organoids could generate branched structures with ductal and end bud-like regions, and we did not observe an effect of Numb-deficiency on cell migration velocity or directional persistence, although the cell packing density was increased. Moreover *in vivo*, other than duct length, we did not observe an effect of Numb-deficiency on other duct dimensions including total duct diameter, lumen diameter, and degree of branching. Therefore, Numb-deficiency unlikely impairs the migration of cells from end buds into ducts. Therefore, we conclude that increased cell packing is the primary cause of ductal elongation impairment in Numb-deficient mammary glands.

Previous studies using a variety of genetically engineered mouse models have identified crucial roles for hormones, hormonal receptors, growth factors, transcription factors, cell signaling and adhesion proteins in ductal development during pubertal mammary gland development (McBryan and Howlin, 2017). To our knowledge, this is the first example of mammary ductal growth impairment caused by changes in cell packing density and suggests a previously unappreciated mechanism that contributes to pubertal mammary gland morphogenesis. Increased cell packing suggests that cells may have reduced tension or stiffness. This notion is supported by our laser ablation data showing reduced cortical tension in cultured Numb-deficient. Moreover, using geometric measurements to calculate relative interfacial and free surface cell tension (David et al., 2014; Winklbauer, 2015), we found that normal tension balance was disrupted in Numb-null mammary epithelial cells. Indeed, the increase in the tension ratio in Numb-deficient cells and reduced flatness of Numb-deficient ducts indicates either an increase in cortical tension at cell-cell contacts, or a reduction in the cortical tension at the free surface. At present, we cannot distinguish these possibilities, however the unbalanced forces would account for the uneven luminal surface and altered cell geometry observed in Numb-deficient mammary ducts.

Cortical tension and cell–cell adhesions are regulated during epithelial morphogenesis and cell rearrangements, as cells need to slide past each, which involves cadherin remodeling, as well as other junctional proteins (Kametani and Takeichi, 2007). Numb is an endocytic adaptor protein and includes E-cadherin and β1-integrin as targets, however the physiologic roles for how Numb regulates these during development and disease is not fully understood. Previous groups observed a decrease in E-cadherin expression and a corresponding increase in EMT markers in the mammary glands when Numb was deleted in basal/myoepithelial cells (Tosoni et al., 2015; Zhang et al., 2016). Unlike these previous reports, we did not observe an increase in EMT characteristics. Instead, we observed enrichment of E-cadherin towards the apical side of the lateral membrane in Numb-deficient mammary glands. These differences are likely due to the different Cre models that target different lineages. However, collectively our data indicate that E-cadherin may be an important Numb target in the mammary epithelium, which likely has cell-type-specific functions. Unlike E-cadherin, we did not observe alterations in β1-integrin localization in Numb-deficient mammary ducts. We propose that Numb is involved in E-cadherin recycling at specific times during mammary gland morphogenesis. One possibility is that Numb controls remodeling of adhesions necessary to switch cells from a dynamic to more stable epithelial phenotype in mammary ducts during morphogenesis. In some epithelia, cadherins undergo basal-to-apical flow in actively migrating cells, but not static cells, which facilitates cells sliding past each other (Kametani and Takeichi, 2007). We speculate that basal-to-apical E-cadherin flow may also facilitate cell sliding as they migrate from end buds and intercalate to elongate a mammary duct. As ducts mature, cells would need to switch from a fluid migratory phenotype to a more stable cuboidal cell shape. The observed apical enrichment in Numb-deficient mammary glands in the present study is consistent with a role for Numb in redistributing apically accumulated E-cadherin. Lateral adhesions would then help maintain normal cell geometry. Along these lines, cellular prestress represents tension within a cell that involves the cytoskeleton through cell junctions of adherent cells, which is necessary to generate forces that provide rigidity to oppose deformation of cell shape (Ingber et al., 2014). Without prestress, cells do not possess the rigidity to maintain cell shapes. Therefore, Numb-deficient cells may lack prestress/rigidity, resulting in cells that can be more easily deformed, which increases cell packing density.

We observed changes in collagen I abundance and organization surrounding ducts and end buds in Numb-deficient mammary glands, suggesting that Numb regulates ECM remodeling. This agrees with other studies where Numb was linked to collagen I expression and renal fibrosis (Zhu et al., 2016). Several reports indicate that ECM composition regulates mammary duct outgrowth during puberty (Hammer et al., 2017; Nguyen-Ngoc and Ewald, 2013; Peuhu et al., 2017). The increase in collagen I fibers surrounding end buds that we observed in Numb-deficient mammary glands could physically impair elongation. How Numb regulates peri-ductal collagen I is presently unknown and should be the focus of future work.

The MMTV-Cre model used in our study targeted cells in both the luminal and myoepithelial compartment as assessed by a tdTomato reporter. However, the cell packing phenotype we observed appears to be exclusive to the luminal epithelial compartment. Previous studies have depleted Numb alone, or along with its homolog NumbL, specifically in the basal/myoepithelial compartment using K5-Cre or K14-Cre mouse lines (Tosoni et al., 2015; Zhang et al., 2016). These studies reported a role for Numb in regulating basal stem cell divisions during development and in lactogenesis during pregnancy (Tosoni et al., 2015; Zhang et al., 2016). Therefore, our study complements these by providing the first analysis that includes Numb-depletion from the luminal population and reveals a previously unappreciated role for Numb in regulating cell tension and epithelial packing density. It is currently controversial whether bi-potent basal mammary stem cells or restricted unipotent cells maintain luminal and myoepithelial lineages in adult mammary gland. However, there is strong evidence for Notch as a key regulator of the luminal lineage. Since Notch is also regulated by Numb in many contexts (Gulino et al., 2010), future studies will examine whether Numb controls Notch-dependent luminal lineage specification in the mammary gland. Curiously, double knockout of Numb and NumbL from basal cells caused an increase in the luminal cell population (Zhang et al., 2016). Tosoni et al. showed that Numb-depletion under a K5 basal promoter caused enlarged, hyper branched ducts in 12-week-old mice (Tosoni et al., 2015). However, Zhang et al. found that using a K14-Cre to deplete Numb that there was a small reduction in ductal outgrowth, with fewer branches and much fewer end buds in eight-week-old glands (Zhang et al., 2016). No such changes in branching or end bud numbers was apparent in our MMTV-Cre model. Our study complements previous work and extends our knowledge of the complexity of Numb functions in mammary morphogenesis. Collectively, this highlights the advantages of studying gene function in epithelial morphogenesis using unique Cre-models to target diverse cell populations at different developmental stages.

## MATERIALS AND METHODS

### Mice

Mice with a conditional floxed Numb(^lox/lox^) allele were derived from Numb(lox/lox)/NumbL(^lox/lox^) double floxed mice obtained from Dr Michel Cayouette (IRCM, Montreal). Each line was backcrossed in an FVB/n background for at least five generations. MMTV-Cre mice in an FVB background were obtained from Dr William Muller (Andrechek et al., 2000) (McGill University, Montreal). Control female mice were either MMTV-Cre or FVB mice, while the Numb-deficient mice were MMTV-Cre;Numb^(fl/fl)^. The fourth inguinal glands were harvested from mice at various points of murine mammary development (four, six and 12 weeks of age) to assess when the effects of Numb-depletion would be most pronounced. To confirm Cre activity in the mammary epithelium, we crossed both control and Numb-deficient mice to a tdTomato reporter strain that has been backcrossed into an FVB/n background (Tran et al., 2014). All procedures involving animals were approved by the McGill University Animal Care Committee.

### Mammary fat pad transplants

Mammary epithelial tissue from FVB-Numb^fl/fl^ mice was isolated as previously described (Halaoui et al., 2017). Epithelial spheroids were infected with Adenovirus-GFP (control) or Adenovirus-Cre-GFP (University of Iowa, Viral Vector Core) to delete Numb, and were transplanted into cleared fourth inguinal mammary fat pads of three-week-old FVB recipients as previously described (McCaffrey and Macara, 2009).

### Immunostaining and imaging

Mouse mammary glands were fixed in either Carnoy's fixative for 4 h at room temperature; or with 4% paraformaldehyde for 16–20 h at 4°C. For immunofluorescence images, paraffin-embedded sections were cut at 6 μm thickness, deparaffinized and stained as previously described (McCaffrey et al., 2012), and primary antibodies applied overnight (with the exception of Numb which was applied for 48 h). Primary antibodies used with dilution and source: Numb (1:400, Cell Signaling Technology, 2576), E-Cadherin (1:500, BD Transduction, 610181), cytokeratin-8 (1:250, DSHB, TROMA-I-c), cytokeratin-14 (1:500, Covance, CLPRB-155P), alpha-smooth muscle actin (1:500, Abcam), Ki67 (1:500, Abcam, ab15580), cleaved-Caspase-3 (1:500, Cell Signaling Technology, 9661), phospho-myosin light chain II (1:500, Cell Signaling Technology, 3671) and ZO-1 (1:500, DSHB, R26.4C-c), β1-integrin (1:100, Abcam, ab30394). The appropriate secondary antibodies conjugated to Alexa488, Alexa546 and Alexa647 were used at 1:750 dilution. In cases of double-labelling with the same species of antibody Tyramide Signal Amplification (TSA) kits (Alexa Fluor 488 tyramide, T20922; Alexa Fluor 555 tyramide, T30954). Confocal imaging was performed using LSM700 from Zeiss with 20X/0.8NA or 40X/1.4 NA lenses and processed using FIJI/ImageJ software (https://imagej.net/Fiji/Downloads) to enhance the brightness and contract uniformly to representative images of the same panel.

### Wholemount mammary gland staining and measurements

Wholemount glands fixed in Carnoy's fixative were stained with Carmine Alum solution to image the whole mammary ductal tree. Glands were imaged on the Zeiss Axiozoom microscope. Images of the Carmine stained wholemounts of the control and Numb-deficient glands were used for the measurement of length traversed by the ducts. Quantification of the ductal elongation for each time point was assessed by using the distal tip of the lymph node as a zero-distance point-of-reference and measuring the distance of the farthest-reaching tip of the ductal tree. The lymph node was used as marker instead of the nipple (in case it was excised during harvesting of the gland), with the edge furthest from the nipple (in the direction of ductal growth) made the reference point. Ducts growing past the lymph node were given a positive length measurement, while those that fell short of it were given a negative length from the reference point. For transplants, the ductal area around the outgrowth was measured since a cleared fat pad has neither a nipple nor lymph node. Whole-gland images at higher magnifications were used to measure the dimensions and shape of TEBs. FIJI/ImageJ was used for measurements.

### Analyses of mammary epithelial cell morphology

To measure the cell packing in the duct, confocal images were taken of control and Numb-deficient sections stained with the luminal marker cytokeratin-8 and basal markers cytokeratin-14/alpha-smooth actin. The number of luminal and basal cells in the duct were counted, and this divided by the length of the duct. To determine the shapes of the luminal cells from fluorescence images, the cell shape was mapped using a segmented line tool in the FIJI/ImageJ software and analyzed using the shape descriptors of area, perimeter and circularity. Area and perimeter provide dimensions of the cell, while circularity is a form of measure of the cell shape. The shape function falls on a scale of 0 to 1, where 1 represents a perfect circle; 0 represents an extended polygon. Animals were numerically coded and investigators were blinded to the genotype during analysis.

### E-cadherin measurements

The fluorescence intensity of E-cadherin was measured along the lateral membrane from apical to basal, and the values for each membrane were used to produce as a plot of E-cadherin fluorescence intensity versus length of membrane in microns. From the raw values of each individual cell the fluorescence intensity was averaged over all the control cells and compared to fluorescent intensity of E-cadherin in all the Numb-deficient cells, and was represented as the average fluorescence intensity for each group over the length of the lateral membrane (in microns).

### Western blot

The level of Numb protein expression was measured by isolating primary epithelial cells (that were not trypsinized to single cells) from control and Numb-/- mice. The cells were lyzed in RIPA buffer supplemented with 50 mM NaF, 5 mM Na_3_VO_4_, protease inhibitor cocktail (Roche, 11836170001) and 1 mM DTT. After a 30-min incubation on ice with complete RIPA buffer, the lysates were cleared by centrifugation at 13,000 rpm for 10 min. After protein estimation 25 ug of cleared lysate was boiled with loading dye at 94°C for 4 min and run on a 10% SDS-PAGE gel. The gel was transferred to a nitrocellulose membrane (Bio-Rad, 1620115) and blocked with 5% w/v milk in TBST. The Numb antibody (1:1000, Cell Signaling Technology, 2576) was applied according to the manufacturer's guidelines. After washing the membrane, a horseradish peroxidase-conjugated anti-rabbit antibody (1:5000, Bio-Rad, 1705046) was applied for 1 h. Blots were washed with TBST three times and developed with the ECL system (Bio-Rad) according to the manufacturer's protocols.

### Organoid culture and analysis of cell migration

Organoids were isolated from mouse mammary glands and plated in an eight-well μ-slide chamber (IBIDI, 82680) on top of a pad of 100% Geltrex™ LDEV-Free Reduced Growth Factor Basement Membrane Matrix (Gibco, A1413202). DMEM/Ham's F-12 (Wisent, 319-075-CL) was supplemented with 1% Insulin-Transferrin-Selenium (Thermo Fisher Scientific, 41400045) and 1% penicillin-streptomycin (Wisent, 450201EL) and 2% Geltrex™.

For tracking experiments, isolated cells were infected with mCherry-histone lentivirus at a multiplicity-of-infection of 10 and cultured for 3 days. The mCherry-positive spheroids were transferred to eight-well μ-slides (Ibidi) and imaged by confocal microscopy for 40 h. mCherry-histone-positive cells were tracked using the TrackMate plugin with ImageJ/FIJI/ImageJ.

### Laser ablation

Primary mammary epithelial cells were isolated from Numb-deficient or control mammary glands and plated in eight-well slides (Ibidi). When cells reached confluence (48 h), they were labeled with CellMask™ Deep Red plasma membrane stain (Invitrogen, C10046) as per the manufacturer's instructions. For laser ablation, a MicroPoint Laser Illumination & Ablation (Andor) system was used: the pulse width for the laser ablation was 3 ns; the laser was ∼70 μJ with ∼25 μJ out of fiber; the laser was set to 30% power; and each pulse was set to five iterations. This system was used in conjunction with a Quorum WaveFX-X1 spinning disc confocal system (40X dry objective lens, 0.85 NA) with images captured every 1.6 s. The recoil distance was calculated as previously described (Liang et al., 2016).

### Birefringence microscopy

Histological sections were stained with Picrosirius Red dye and ducts from control and Numb-deficient sections were imaged using Zeiss Axiovert 200M Fully Automated Inverted Microscope with an objective 20X lens (NA=0.80). Brightfield and polarized light images were taken and analyzed using FIJI/ImageJ. To quantify the overall amount of collagen present, the total collagen was measured from the polarized light images and normalized to the perimeter of the duct to generate a ratio. To determine the density of collagen bundles around the duct a custom macro in FIJI/ImageJ was used to quantify the pixels in the color range of red, orange, yellow and green. This macro translated colors from RGB into the HSB color space, and boundaries were set for red (0–14), orange (15–44), yellow (45–74) and green (75–150) (example shown in Fig. S3A). The pixels were binned from the polarized light images into these four color categories. The pixel percentage was calculated for each color and plotted. The macro is available upon request to the authors.

### Statistical analysis

Comparisons of multiple means was performed by one-way ANOVA (Bonferroni) for the multiple comparison of ductal lengths between six-week control, Numb-deficient. Alpha=0.05 was used to determine statistical significance. Unpaired Student's *t*-test was used to compare to sets of means with a confidence interval of 95% using Excel or GraphPad Prism.

## Supplementary Material

Supplementary information
