# Supplementary material for: Numb regulates cell tension required for mammary duct elongation
Source: Biol Open. 2019 Apr 29;8(5):bio042341. doi: 10.1242/bio.042341 (PMC6550071; doi:10.1242/bio.042341)
Supplement: Supplementary information [file biolopen-8-042341-s1.pdf]

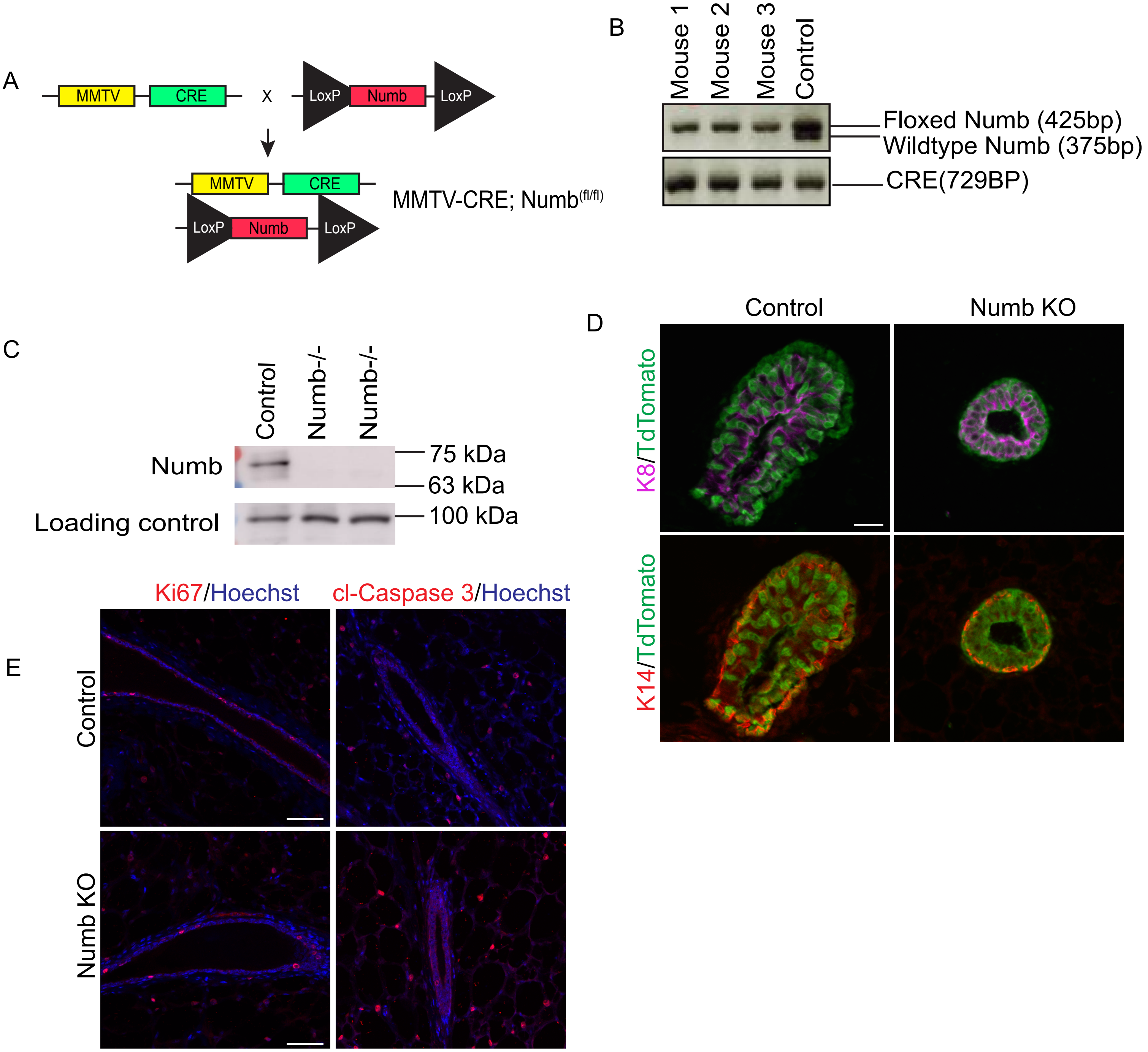

Fig. S1. Expression of Numb, Cre, Ki67, and cleaved Caspase 3. (related to Figure 1)
A) A model of the strategy used to produce conditional Numb knockout in mouse mammary glands. B) Genotyping indicating the presence of floxed Numb (423bp) in conjunction with Cre expression (729 bp) for three different mice; controls indicate a positive control (floxed Numb, 425bp) and a negative control (wildtype Numb, 375bp). C) Western blot of Numb protein expression in control and Numb-/- samples. The loading control is a non-specific band. Levels of Numb were measured using FIJI/IMAGEJ and normalized to the corresponding band of the loading control. D) Fluorescence images of control and Numb-deficient mice crossed with Gt (Rosa)tdTomato to generate mice that express tdTomato upon Cre expression. Immunostained performed with tdTomato (green), the luminal marker cytokeratin 8 (CK8, magenta) and the basal marker cytokeratin 14 (CK14, red). E) Fluorescence images of Numb-deficient and control ducts immunostained with Ki67 (proliferation) and cleaved-Caspase 3 (apoptosis). Scale bars: C, 20µm; D, 20µm

Biology Open • Supplementary information

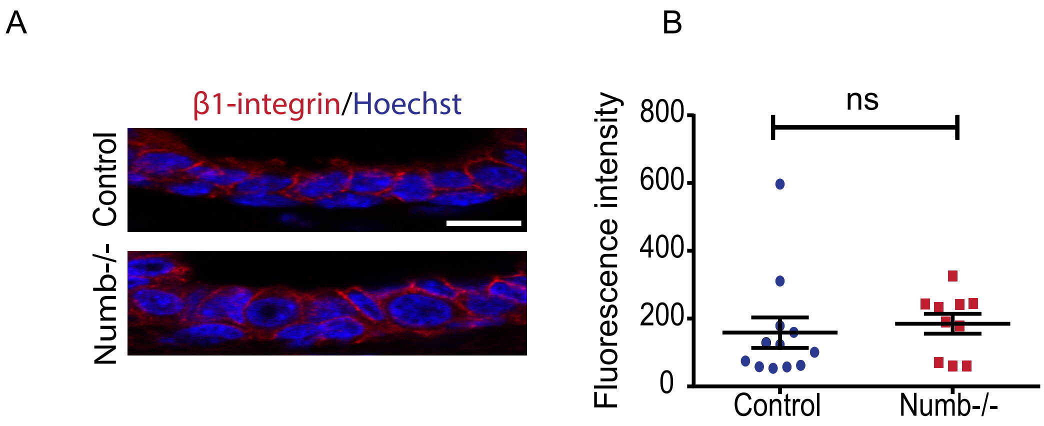

Fig. S2. Numb does not regulate β1-integrin localization. (related to Figure 6)
A) Fluorescence images of control and Numb-deficient ducts immunostained with β1-integrin shows no change localization of accumulation on the membranes of control or Numb-deficient cells. B) Scatter plot of the average β1-integrin fluorescence intensity measured across a length of control and Numb-deficient ducts.

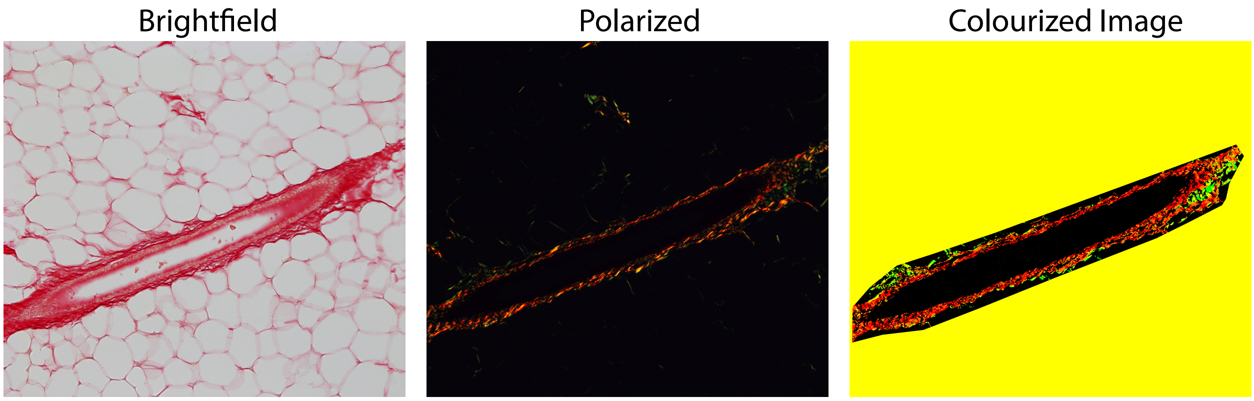

Fig. S3. Validation of colorization for polarized light image analysis. (related to Figure 8)
A representative image of a colorized image (right) generated by a macro used to re-create the colors of the corresponding polarized light microscopy image (centre). The macro was used to produce pixel numbers for each color (red, orange, yellow, green) to quantify polarized light images. Scale bars: 100μm
